# Identification of skin signs in human-trafficking survivors

**DOI:** 10.1016/j.ijwd.2021.09.011

**Published:** 2021-10-02

**Authors:** Raaga Rambhatla, Marielle Jamgochian, Cristina Ricco, Rohan Shah, Hira Ghani, Channi Silence, Babar Rao, Arianne Shadi Kourosh

**Affiliations:** a Rutgers Robert Wood Johnson Medical School, Piscataway, New Jersey; b Rutgers, New Jersey Medical School, Newark, New Jersey; c New York Institute of Technology College of Osteopathic Medicine, Old Westbury, New York; d Massachusetts General Hospital Department of Dermatology, Boston, Massachusetts; e RWJMS, Center for Dermatology, Somerset, New Jersey; f Harvard Medical School, Boston, Massachusetts

**Keywords:** Human trafficking, sex trafficking, labor trafficking, tattoo branding

## Abstract

Human-trafficking survivors suffer significant physical, mental, and social health consequences, prompting them to seek health care services. Although there is research regarding identification protocols for human-trafficking victims, there is no framework outlining the dermatologic patterns of survivors of human trafficking. We sought to identify the dermatologic signs reported in human-trafficking victims to create a framework for dermatologists and the broader medical community to appropriately screen patients at risk. After screening 577 pertinent records in the PubMed and Google Scholar databases for information about the physical signs of human trafficking in health care, 10 final studies were selected. Significant findings of rashes and brandings, such as tattoos, were more likely in sex-trafficked patients, whereas burns, injuries, and deep cuts were more likely to be found in labor-trafficked patients. This review outlines important identification guidelines that dermatologists and the broader medical community can use to recognize victims and take appropriate action while also raising awareness of human trafficking as an emerging public health issue.

**What is known about this subject in regard to women and their families?**

• Women and children are the predominant group of victims trafficked across the globe.
• Sexual exploitation of women is the most prevalent category of human trafficking, followed by forced labor trafficking.
• Fifty percent to 80% of trafficked individuals seek health care while being exploited.
• Although protocols are in place to identify victims of human trafficking in the emergency department, there is limited research on the patterns of dermatologic presentations needed for the timely identification and intervention of those affected.

**What is new from this article as messages for women and their families?**

• Tattoos, branding, rashes, and bruising are typically seen in victims of sex trafficking, whereas cuts, burns, and skin injury are more likely found on victims of labor trafficking.
• Dermatologists can be leading providers in recognizing cutaneous manifestations of human trafficking to identify victims sooner and reduce harm in this population.
• Skin examination is an important component of a comprehensive patient evaluation necessary to identify human-trafficking survivors.

Alt-text: Unlabelled box

## Introduction

Human trafficking is a human rights violation affecting millions of individuals, with the use of force, deception, or coercion to recruit and exploit individuals for labor and commercial sex acts. Trafficking does not discriminate by age, race, gender, or nationality; however, vulnerable groups at increased risk of becoming trafficked include incarcerated or formerly incarcerated individuals, foster and runaway youth, victims of sexual abuse, women, and LGBTQ+ individuals (Armstrong et al., 2019). According to the United Nations (United Nations Office on Drugs and Crime, 2020), women are the most trafficked victims across the globe. Human trafficking is an emerging public health issue; however, there is limited research and expertise in the medical field on this topic.

Contrary to popular belief, trafficking is not synonymous with smuggling. Trafficking does not require crossing state or national borders, and an estimated 83% of trafficked individuals in the United States are citizens (Shandro et al., 2016). Many people are trafficked within their own homes. An estimated 50,000 people are trafficked in the United States each year. Globally, 20.9 million are victims of forced labor, including 4.5 million victims of forced sexual exploitation (Shandro et al., 2016). Most cases go unreported due to language barriers and fear of traffickers and law enforcement. Educating medical staff on indicators of human trafficking can assist with identifying and providing resources for victims of trafficking.

Trafficking often takes two forms: labor and commercial sex. Labor trafficking includes domestic servants, agricultural farmworkers, and factory workers. Victims are often exposed to dangerous and inhumane conditions and can present with occupational injuries and dermatitis. When minors (age <18 years) engage in commercial sex, it is considered sex trafficking, regardless of coercion or fraud. According to the Global Report on Trafficking, 50% of trafficked persons were victims of sexual exploitation, 38% forced labor, and 6% to 7% were involved in criminal activity (United Nations Office on Drugs and Crime, 2020). Women being trafficked for sexual exploitation was the most prevalent category of trafficking among all major geographic regions surveyed.

Many trafficked people suffer physical, sexual, and psychological abuse. In the United States, research suggests that 50% to 88% of identified trafficked persons seek health care while they are being exploited (Armstrong et al., 2019). There is limited research on the patterns of physical examination findings that have historically led to the identification of and intervention with affected individuals. It is unknown how often these patients are seen in specialty care settings (e.g., dermatology), but expertise in the physical diagnosis offered by a dermatologist and awareness of characteristic dermatologic signs as suggested by current evidence could be helpful to those on the frontlines. Unusual patient behavior, skin signs, including bruises, tattoos, and physical injuries, and submissive/rehearsed responses should be recognized by physicians and investigated further.

## Methods

A comprehensive narrative review was conducted using the PubMed and Google Scholar databases (January 2010–December 2020) to identify relevant articles using the Preferred Reporting Items for Systematic Reviews and Meta-Analyses guidelines (Page et al., 2021). The initial search was performed in June 2021, after which the titles and abstracts were screened for inclusion criteria by two independent reviewers (M.J., R.R.). Full texts of the relevant articles were then reviewed by the same two reviewers (M.J., R.R.) to ensure they met the inclusion criteria. Any disagreements were resolved by a third reviewer (H.G.). Additional relevant articles were included from the bibliography of selected articles found during our database searches. Subsequently, pertinent variables, such as type of study, type of human trafficking, patient demographics, focus of the study, and signs of physical/cutaneous and psychological abuse, were extracted from each study.

### Search criteria

Combinations of search terms were run in both databases. We used the following search string to identify relevant articles.

#### Google Scholar

trafficking identification physical **OR** sex **OR** labor **OR** education **OR** care **OR** abuse: 65 results
AND
trafficking, identification, care physical, **OR** labor, **OR** sex: 78 results
PubMed
(((labor trafficking) **OR** (sex trafficking)) **AND** (skin)) **AND** (care): 15 results
AND
((labor trafficking) **AND** (identification)) **AND** (care): 21 results
AND
(((sex trafficking) **AND** (identification)) **AND** (care): 60 results
AND
((abuse) **AND** (identification)) **AND** (physical)) **AND** (care)) **AND** (emergency): 81 results
AND
(((exploitation) **AND** (identification)) **AND** (human)) **AND** (care) **AND** (emergency): 35 results
AND
(labor trafficking) **AND** (identification): 215 results

With respect to the medical literature, we limited our search to peer-reviewed articles published in the last 10 years. We assessed article quality, study context and design, and outcomes. Inclusion criteria included studies on PubMed and Google Scholar, written in the English language, and those that specifically mentioned physical signs of human trafficking in health care settings. We limited our designs to surveys, cohort studies, observational studies, and case reports/reviews to obtain and pool patient data. The exclusion criteria involved studies that were not accessible for full-text review, excluded discussion of human-trafficking identifiers, and comprehensive reviews. Although reviews of human trafficking provided useful background and identification protocol summaries, they had limited information pertaining to specific dermatologic signs.

**Fig. 1:**
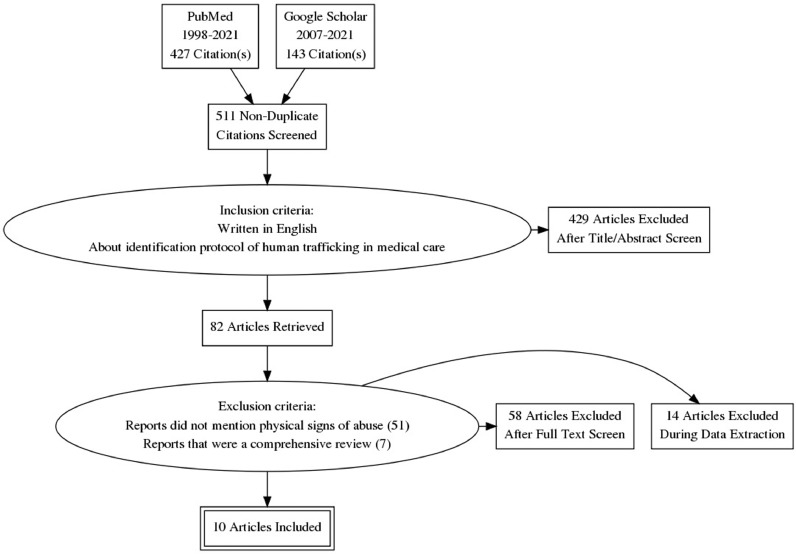
Preferred Reporting Items for Systematic Reviews and Meta-Analyses flowchart outlining the inclusion and exclusion criteria for this review.

A total of 577 records were generated using the search terms, of which 213 studies were extracted. After screening the titles/abstracts and full texts for the inclusion criteria, 10 studies were shortlisted for our literature review (Fig. 1).

## Results

Of the 10 studies included (Table 1), two were surveys of providers of services for trafficked individuals, and 808 total providers were surveyed. Gerassi et al. (2021) surveyed participants (N = 86) to identify the most observed indicators of trafficking using a 5-point Likert scale. Participants were in health care (n = 19), prostitution/trafficking services (n = 14), domestic violence/sexual assault services (n = 13), youth services (n = 8), and juvenile justice (n = 5). The most common indicators selected were mental health symptoms of “depression (M = 3.82); low self-esteem (M = 3.59); anxiety (M = 3.55); low levels of interpersonal trust (M = 3.52); sense of fear (M = 3.36); feelings of shame or guilt (M =3.34); isolation from family, friends, and communities (M = 3.3); and fear/distrust of law enforcement (M = 3.80).” Less common indicators included the presence of tattoos or branding (M = 1.89) and physical evidence of torture (M = 2.07).

**Table 1:** Dermatologic signs observed in the studies included in this review

Study	Location	Article type	Type of trafficking	Sample	Dermatologic signs
Zimmerman et al., 2008	Europe, United Kingdom	Survey	Sex	192 women and girls accessing posttrafficking services in Europe and the United Kingdom	Rashes, itching, or sores: 54 of 192 (28.13%)
Oram et al., 2016	England	Cross-sectional survey	Sex, labor	150 men, women, and children using posttrafficking services in England	Rashes, itching, or sores: 28 of 150 (18.6%)
Goldberg et al., 2017	United States	Retrospective cohort	Sex	41 male and female children referred for postsex trafficking services	Of those with documented physical examinations, 14 of 38 had cutaneous findings (e.g., bruising) that were incidental or self-inflicted; 8 of 38 (21%) had at least 1 tattoo. Patient-reported self-injurious behaviors in 4 of 41 patients.
Gibbons et al., 2016	United States	Case report	Sex	1	Forehead abrasion
Nguyen et al., 2018	United States	Case series	Sex and labor	6 cases (5 females, 1 male on an inpatient psychiatry service)	One female patient with consistent, severe, nonsuicidal, self-injurious behavior; second female with “multiple tattoos across her body consistent with known forms of human trafficking branding”
Mumma et al., 2017	United States	Observational cohort	Sex	10 women who presented to the emergency department and were identified as victims of sex trafficking	One of 10 presented with CC of trauma/injury; this was not described in further detail. Physical examination findings not described in detail.
Kiss et al., 2015	Cambodia, Thailand, Vietnam	Observational cross-sectional study	Sex and labor	1015 men, women, and children accessing posttrafficking services	Skin problems (n = 127; 12.5%); deep or long cut (n = 115; 11.3%); very bad burn (n = 31; 3.1%); skin damage or injury (n = 85; 8.4%)
Lederer et al., 2014	United States	Survey	Sex	107 female sex-trafficking survivors	27.4% of respondents reported dermatologic issues (29 of 107)
Schwarz, 2017	United States	Cross-sectional survey of providers treating trafficked persons	Sex, labor	722 providers treating trafficked persons in the U.S. Midwest	Presence of tattoos or branding was reported as more likely in those at risk of sex trafficking than in those at risk of labor trafficking
Gerassi et al., 2021	United States	Survey of providers treating sex-trafficking victims	Sex	86 service providers for trafficked people	5-point Likert scale: “presence of tattoos or branding (M = 1.89) and physical evidence of torture (M = 2.07)”

In Schwarz (2017), 722 service providers (171 medical, 149 legal/law enforcement, 90 nonprofit, 42 social service 42, and 270 foster care) were asked “what conditions are you most likely to see among individuals who may be at risk for labor or sex trafficking?” The most commonly noted findings in human-trafficking victims were psychiatric concerns, drug or alcohol abuse, and untreated sexually transmitted infection (STI). The presence of tattoos or branding was reported as more likely to be seen in those at risk of sex trafficking than in those at risk of labor trafficking. The two studies of service providers were heterogeneous in measured outcomes and thus could not be compared directly. 

Eight studies described physical findings in human-trafficking victims that may be apparent during a dermatologic examination from a total of 1522 patients. These studies also described other findings that are common indicators of human trafficking. In data pooled from Zimmerman et al. (2008) and Oram et al, (2016), frequently identified dermatologic signs included rashes, itching, or sores in 82 of 342 patients (24%), more frequently found in victims of sex trafficking. Deep or long cuts (115 of 1015 patients), very bad burns (31 of 1015 patients), or skin injuries (85 of 1015 patients) were more frequently found in victims of labor trafficking. Labor trafficking in the fishing and factory industries had the most thoroughly documented incidence of skin injury (Kiss et al., 2015). Nonspecific dermatologic symptoms were reported in 27.4% of the 107 interviewed patients (Lederer et al., 2014). Forehead abrasion was documented as a cutaneous finding in a case report of a trafficked patient (Gibbons et al., 2016). Mumma et al. (2017) described 10 patients admitted to the emergency department who were positively identified as victims of trafficking through a screening protocol, with one patient reporting trauma/injury. However, the physical examination findings were not described in detail.

Nguyen et al. (2018) described skin signs in two of six patients: one with repeated self-injurious behavior and another with no cutaneous injury but the presence of multiple tattoos, consistent with branding. In Goldberg et al. (2017), cutaneous injury and bruising was noted in 14 of 38 survivors of domestic child sex trafficking, and 8 of 38 survivors had tattoos. Tattoos are frequently cited as possible signs of trafficking (Tracy et al., 2017); however, there is little literature information on the types or description of tattoos found in human-trafficking victims and survivors. The frequency of tattoos in trafficked populations is unknown; however, the discovery of a tattoo in one of the categories described that is of poor quality rather than professional appearance—especially if found on a minor—should prompt the physician to inquire about the nature of the tattoo (Fang et al., 2018). The studies were heterogeneous in their outcomes and most could not be directly compared.

## Discussion

Our goal was to identify and outline commonly reported physical signs and presentations of human trafficking in health care settings to assist with more timely identification of and intervention with affected persons. To our knowledge, there are no standardized protocols with identification recommendations and screening strategies currently available to health care professionals, despite the prevalence and widespread geographic distribution of human trafficking. Although there is some research on the risk factors and indicators of victims in the emergency department and primary care setting (Greenbaum, 2016), there is no such framework for physicians in specialty care, specifically the physical examination findings and tattoo-related features that can be informed and guided by dermatologists. Our hope is that every physician will be able to screen for and detect unusual patient signs and behaviors of human trafficking. Thus, the purpose of our research was to synthesize all available studies that highlight the specific indicators of potential abuse/trafficking detectable from a physical examination, in conjunction with other behavioral and health conditions. Taken together, the findings of this review offer physicians, residents, nurses, and all medical staff important identification guidelines with the lens of dermatology that can be used to recognize victims, provide appropriate care, and take action. For example, tattoos are a visible risk factor for hepatitis C, so noting a tattoo should indicate and prompt disease screening (Carney, 2013). The indicators in this review can also guide future research into the need for dermatology education and resident training in victim identification and screening.

Before implementing policies or practice guidelines, it is important to understand that human trafficking is relatively common, and providers have likely missed opportunities to identify such patients in practice. Many physicians are largely unaware of and misinformed about the scope and complexity of the issue (Shandro et al., 2016). This knowledge gap is underscored by the limited original data from surveys and observational studies available during our literature search. Although this review is aimed at dermatologists, the studies included are targeted toward other types of care, including emergency, primary, and posttrafficking care. Most of the current literature regarding human-trafficking identification focuses on common presentations obtained from the psychiatric and medical history, including depression, substance abuse, and sexually transmitted diseases, but our review focuses solely on the physical signs of abuse to underline the necessity of including a skin examination in any health care setting where a suspected patient is being screened. Dermatologists can lead the way for the medical community in performing the physical examination as part of a comprehensive evaluation of the patient.

The most common cutaneous signs described in the literature include deep or long cuts, itching or sores, and the presence of tattoos or branding. Rashes and brandings were more likely to be found in sexually trafficked patients, and burns and deep cuts were more likely to be found in labor-trafficked patients (Table 2). An untreated STI was another common finding with cutaneous manifestations. The most prevalent noncutaneous indicators included depression, low self-esteem, anxiety, feelings of shame, and a distrust of medical personnel and law enforcement. One review study noted that the most common chief complaint to the emergency department was trauma or injury (Shandro et al., 2016).

**Table 2:** Commonly observed physical signs in survivors of sex and labor trafficking

Type of trafficking	Associated physical signs
Sex	Tattoos, branding, rashes, bruising, self-injurious behavior
Labor	Burns, deep cuts, skin damage/injury

**Fig. 2:**
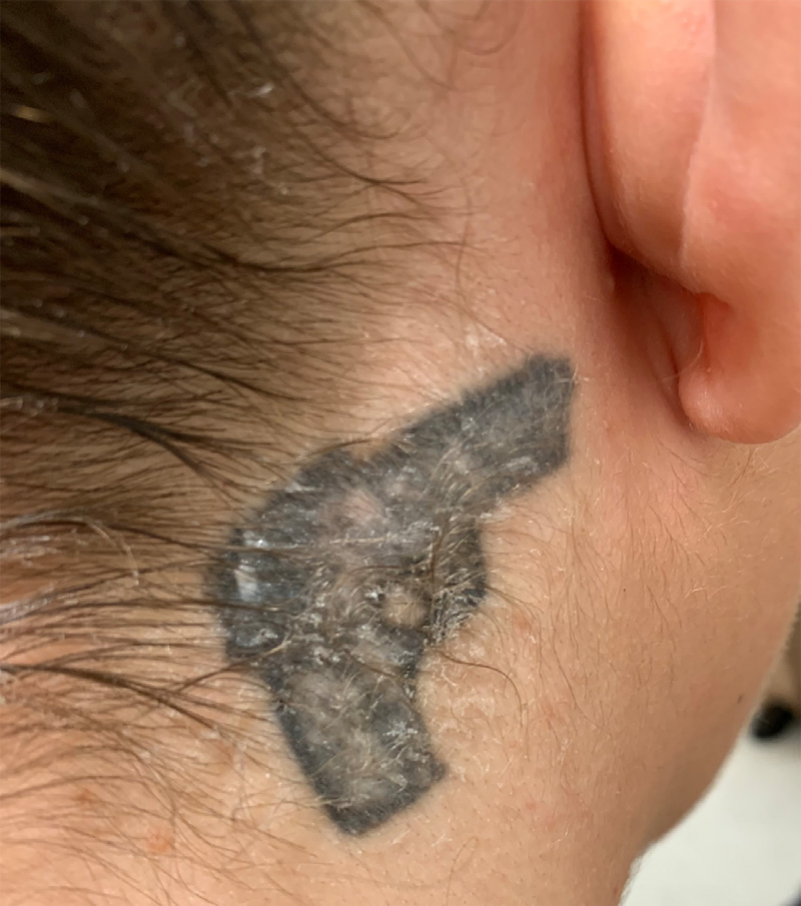
Example of a tattoo on a human trafficking survivor. Note the poor quality and location of the tattoo on the neck.

The cutaneous sign of branding and tattoos has been a repeating theme in the literature on human trafficking, yet much ambiguity remains in translating this finding into a mechanism by which clinicians can identify trafficked patients. First, the proportion of the general population that has tattoos is high, thus creating figurative background noise in the identification of relevant tattoos. Second, the frequency and common motifs of tattoos in trafficked populations remain unknown. Thus, future investigation into the specific brandings of trafficked patients may prove valuable in identifying members of this population. Here we provide a photograph that a trafficking survivor treated by one of the authors consented and volunteered to share to aid the medical community in identifying and intervening for others who are affected (Fig. 2). The poor quality of the tattoo can be attributed to its “homemade nature,” as Fang et al. (2018) describe. The neck, due to its visibility, and the depiction of weapons, is highly associated with gang/crime tattoos. This may suggest that gangs and crime rings may be common perpetrators of trafficking (Korman, 1995).

The dermatologist is critical in improving diagnostics and care for trafficked patients. According to our findings, cutaneous manifestations are a leading sign of trafficked individuals. Although there is an emphasis in the literature on the psychiatric and mental trauma that trafficked patients experience (Oram et al., 2016), our findings imply that, in addition to the emphasis placed on mental health during the physical examination, a skin examination is equally important in discerning physical abuse. In addition, due to a lack of comfort in the physician–patient interaction, many trafficked patients do not return to the same provider for a second visit (Macy et al., 2012). This makes it more difficult to maintain a clinical relationship with the patient. As a result, it is even more important to be comprehensive during the initial visit, especially when the dermatologist suspects that their patient is, or has previously been, trafficked.

The physician should thoroughly perform the physical examination and document all signs of injury, abuse, tattoos, piercings, lesions, and surgical incisions. Given the prevalence of mental health and behavioral manifestations of trafficking, the physician should also document if any signs of anxiety, depression, posttraumatic stress disorder, drug abuse, and suicidal ideation are addressed or recognized during the examination. If the patient is accompanied by a companion, the apparent power dynamics and patient demeanor should be observed as well. Often, the health complaints leading victims to seek medical care are genitourinary conditions and traumatic injuries, such as sexual assault, pelvic pain, STI, urinary tract infection, abortion, or other pregnancy-related issues.

When patients present with such complaints, the provider should become alert to the possibility of trafficking and screen accordingly. If the findings are consistent with confirmed or suspected human trafficking, the physician should take further action in accordance with the patient's safety and goals for the encounter. The immediate obligation is to provide medical intervention and treatment for the chief complaint. In terms of further assistance, providers should keep in mind that each situation is unique. Some victims may not be ready to leave their situation, and others may fear law enforcement (Armstrong et al., 2019). To maintain trust and rapport between the physician and patient, a physician may choose to provide victims with the National Human Trafficking Resource Center hotline and other local resources.

Because the reviewed papers evaluate individuals who are mostly seeking posttrafficking services, it is difficult to determine which cutaneous signs are apparent in those who are currently being trafficked. A case-control study would further be able to determine the differences in the presence of skin signs and symptoms in trafficked persons compared with the general population. Other limitations from our study stem from the lack of literature and standardized documentation on this topic. For instance, many evaluated studies did not distinguish between labor and sex trafficking, which affected our ability to effectively separate the two different trafficking types and generate specific guidelines tailored for each.

This review is aimed to generate discourse in the dermatology community on this emerging public health issue because many of the signs and symptoms of trafficked patients are cutaneous in nature. However, many of the reviewed articles in this study were directed toward other types of practicing physicians, such as emergency and primary care providers; thus, the specificity and thoroughness of the descriptions were often not in the standard or fashion of dermatology. Thus, although we hope our findings provide a foundation for all care providers, this study is limited by the sparsity and level of thoroughness in the descriptions of skin manifestations documented to date.

## Conclusion

With this review, we hope to generate discourse within the dermatology and broader medical community on trafficking as an emerging global public health issue; we believe that, as physicians, we are only beginning to scratch the surface. The United Nations's Global Trafficking Report has been in print since 2009, detailing the extent of the atrocities that occur every day around the globe, yet a national, standardized protocol for identifying victims of human trafficking has not yet been implemented across health care institutions.

Given that these patients often go through the health care system unrecognized and that cutaneous manifestations are among the major signs of trafficked individuals, we call upon dermatologists in particular to pay close attention to this topic. Dermatologists can lead the medical community in identification, systematic study, and the development of registries that could enable more accurate and organized characterization of skin manifestations and thus shape future guidelines and curricula.

We have compiled in this review the relatively few known signs to aid in skin examinations of individuals who may be trafficked as an initial step. We hope others can expand the documentation and analysis of dermatologic observations in trafficking. Example photographs of brandings and common locations on the body will be especially helpful to providers when taking histories and performing examinations. We hope that training in the signs of trafficking for residents and students will become incorporated into medical curricula and that raising awareness in the medical community will allow physicians to more effectively impact the lives of these patients.
